# Neutrophil Inflammatory Response Is Downregulated by Uptake of Superparamagnetic Iron Oxide Nanoparticle Therapeutics

**DOI:** 10.3389/fimmu.2020.571489

**Published:** 2020-12-09

**Authors:** Gustavo Garcia, Min-Ho Kim, Vasilios Aris Morikis, Scott I. Simon

**Affiliations:** ^1^ Department of Biomedical Engineering, University of California, Davis, Davis, CA, United States; ^2^ Department of Biological Sciences, Kent State University, Kent, OH, United States

**Keywords:** neutrophil recruitment, nanoparticle, iron oxide, inflammation, neutrophil degranulation, mechanosignaling, immunosupression, innate immunity

## Abstract

Superparamagnetic iron oxide nanoparticles (SPION) are employed as diagnostics and therapeutics following intravenous delivery for the treatment of iron deficiency anemia (IDA) in adult patients with chronic kidney failure. Neutrophils are the first defense against blood borne foreign insult and recruit to vascular sites of inflammation *via* a sequential process that is characterized by adhesive capture, rolling, and shear resistant arrest. A primary chemotactic agonist presented on the glycocalyx of inflamed endothelium is IL-8, which upon binding to its cognate membrane receptor (CXCR1/2) activates a suite of responses in neutrophils. An early response is degranulation with accompanying upregulation of β2-integrin (CD11/CD18) and shedding of L-selectin (CD62L) receptors, which exert differential effects on the efficiency of endothelial recruitment. Feraheme is an FDA approved SPION treatment for IDA, but its effect on the innate immune response of neutrophils during inflammation has not been reported. Here, we studied the immunomodulatory effects of Feraheme on neutrophils freshly isolated from healthy human subjects and stimulated in suspension or on inflammatory mimetic substrates with IL-8. Cells treated with Feraheme exhibited reduced sensitivity to stimulation with IL-8, indicated by reduced upregulation of membrane CD11b/CD18 receptors, high affinity (HA) CD18, and shedding of CD62L. Feraheme also inhibited N-formyl-Met-Leu-Phe (fMLP) induced reactive oxygen species production. Neutrophil rolling, arrest, and migration was assessed in vascular mimetic microfluidic channels coated with E-selectin and ICAM-1 to simulate inflamed endothelium. Neutrophils exposed to Feraheme rolled faster on E-selectin and arrested less frequently on ICAM-1, in a manner dependent upon SPION concentration. Subsequent neutrophil shape change, and migration were also significantly inhibited in the presence of Feraheme. Lastly, Feraheme accelerated clearance of cytosolic calcium flux following IL-8 stimulation. We conclude that uptake of Feraheme by neutrophils inhibits chemotactic activation and downregulates normal rolling to arrest under shear flow. The mechanism involves increased calcium clearance following chemotactic activation, which may diminish the efficiency of recruitment from the circulation at vascular sites of inflammation.

## Introduction

Superparamagnetic iron oxide nanoparticles (SPION) are used as contrast agents for magnetic resonance imaging (MRI) in the liver, central nervous system, gastrointestinal system, and in macrophages (1). SPION have also found clinical use as an FDA approved treatment of cancer (i.e., Nanotherm) and iron deficiency anemia (IDA) in patients with chronic kidney disease (CKD) (i.e., Feraheme) (1). While SPION have been used for various clinical applications, a primary consideration for any biomaterial based therapeutic is the host immune response elicited by the interaction of the biomaterial with blood cells. The increased use of SPION has raised concerns over their safety, with several formulations showing cytotoxic and immunotoxic effects (2). This motivated the current study to examine the systemic effects of Feraheme on neutrophils in human blood.

The innate immune system is by design able to recognize and eliminate foreign pathogens and particulate matter through pattern recognition receptors and opsonin receptors that sound the alarm upon detection of danger signals (3). Opsonization involves the binding of molecules such as antibodies or proteins of the complement system that enhances recognition and removal *via* endocytosis and phagocytosis of foreign matter by innate immune cells (4). As nanoparticles enter the blood stream, complement and other proteins immediately adsorb to the particle surface forming a corona (5). Physiochemical properties of nanoparticles have a direct influence on the composition and formation of the protein corona in such a manner to impact particle-cell interactions (6). Induction of immune hypersensitivity reaction in patients prompted FDA to cancel clinical use of the SPION Feridex and GastroMARK (7, 8). Further, studies have shown that SPION can induce oxidative stress and cell damage *in vitro* and in animal models (9, 10). Upon binding to the plasma membrane and endocytosis, nanoparticles can induce a variety of functional responses in neutrophils such as reactive oxygen species (ROS) and chemokine production that vary with different SPION formulations. While some SPION can induce these immune responses, others exert an inhibitory effect, thereby highlighting composition as a key factor when considering nanoparticle use as a therapeutic (2, 11). Conventional wisdom is that nanoparticle clearance is mediated by macrophages; however, recent studies indicate that neutrophils also play a key role in particle clearance and this process has been exploited to deliver therapeutic nanoparticles to tumors for cancer therapy (12, 13). Current literature on the immune effects of SPION have largely focused on monocytes and macrophages. This has resulted in an incomplete understanding of the full scope of SPION immune effects on neutrophil function (14).

Neutrophils are the most common leukocyte in blood circulation and are essential first responders during inflammation. Thus, intravenous administration of nanoparticles is likely to result in frequent neutrophil-particle interactions. Capture of SPION onto the plasma membrane and subsequent endocytosis can perturb the inflammatory response and recruitment of neutrophils to sites of inflammation. Dysregulation of neutrophil activation and recruitment is implicated in various autoimmune and inflammatory diseases (15). Failure of neutrophils to normally adhere to the endothelium and become activated can cause severe impairment of host defense against pathogens, while inappropriate levels of cell recruitment and activation leads to chronic inflammation and tissue damage (16, 17). There are reports of the capacity for coated (Polyacrylic acid) and non-coated iron oxide nanoparticles and metal oxide nanoparticles TiO2, CeO2, and ZnO, to induce ROS production and activate neutrophil degranulation (18, 19). Excessive release of neutrophil granules, which contain chemokine and integrin receptors, elastase, collagenase, and myeloperoxidase, along with overproduction of ROS can lead to inflammatory tissue injury (20, 21). Uptake of gold nanoparticles has been linked to endoplasmic reticulum stress and cleavage of cytoskeletal proteins in human neutrophils leading to apoptosis (22). Further, nano and micro particles have been reported to inhibit the recruitment of neutrophils from the circulation to sites of inflammation in mice and to cause a reduction in neutrophil attachment to endothelial monolayers in vascular mimetic flow channels (23, 24). The fine balance between the protective functions of neutrophils that maintain immune competence versus exuberant response that can result in tissue and organ damage has prompted the current studies on neutrophil activation and inflammatory recruitment in the presence of Feraheme. Intravenous infusion with Feraheme for delivery of complexed iron is FDA approved at a higher single dose than other products on the market (25). A typical treatment regime consists of two 510-mg doses separated by 5–8 days as compared to other products that require between 5 and 10 individual doses, thereby limiting the need for repeated infusion which typically lowers patient compliance. Feraheme also is approved for infusion at higher rates than other iron products with comparable safety (25, 26). The increased application of Feraheme in anemia patients who can receive frequent intravenous injections as a course of therapy highlight a need to understand the effects of SPION on neutrophils and their capacity to maintain normal immunosurveillance.

In the current study, we examined the effects of Feraheme on neutrophil degranulation and alterations in receptor expression and conformation on the plasma membrane. We hypothesized that neutrophil uptake of Feraheme in blood alters the normal process of adhesion receptor activation, shear stress resistant neutrophil rolling to arrest and subsequent shape change that precedes cell migration. Vascular mimetic flow channels were employed to assess the kinematics of neutrophil interactions on a substrate of recombinant E-selectin and ICAM-1 in an established model of endothelial inflammation. Feraheme in suspension inhibited neutrophil activation and degranulation induced by IL-8 stimulation, resulting in alteration in the expression of adhesion molecules necessary for the efficient recruitment on inflamed endothelium.

## Materials and Methods

### Small Molecules, Antibodies, and Other Reagents

Monoclonal antibodies for flow cytometric detection of high affinity β2-integrin (mAb24), L-Selectin (Dreg-55, Dreg-56), CD11b (M170), CD18 (1B4), CD66b (G10F5) CD11a (HI111), PSGL-1 (PL-2, KPL-1), CXCR1(8F1/CXCR1), and CXCR2 (5E8/CXCR2) along with antibodies that block CD11b function (Mac-1 blocking, M1/70), fixation buffer, and IL-8 were purchased from Biolegend (San Diego, CA). Recombinant human ICAM-1-IgG and E-Selectin-IgG produced as Fc chimeric constructs were purchased from R&D Systems (Minneapolis, MN). Adenosine A_2A_ receptor agonist CGS-21680, N-formyl-Met-Leu-Phe (fMLP), and ROS indicator Dihydrorhodamine 123 were purchased from Millipore Sigma (Burlington, MA). Adenosine A_2A_ receptor antagonist ZM 241385 was purchased from Tocris Bioscience (Minneapolis, MN). Feraheme (AMAG Pharmaceuticals, Waltham, MA) was purchased from the UC Davis Medical Center Pharmacy.

### Human Neutrophil Isolation

Whole blood was obtained from healthy donors consented through a University of California, Davis institutional review board protocol #235586-9. Neutrophils were isolated from whole blood *via* negative enrichment using EasySep™ direct human neutrophil isolation kit purchased from StemCell Technologies as per manufacturers instruction (Cambridge, MA). Some studies employed a percoll gradient separation using Polymorphoprep® as per manufacturers instruction (Fresenius Kabi). Briefly, for gradient based isolation, whole blood is layered on an equal volume of Polymorphoprep solution and centrifuged at 760g for 30 min at 25°C. The neutrophil layer is collected and washed in Phosphate-buffered saline (PBS) before resuspension in HBSS buffer containing 0.1% human serum albumin (HSA) without Ca^2+^ and Mg^2+^ and kept at 1 × 10^7^ cells/ml on ice after isolation and prior to experimentation. For EasySep neutrophil isolation, whole blood was diluted 1:1 with PBS then 100 µl of isolation cocktail and magnetic RapidSpheres each were added and incubated for 5 min. Cells were then placed in the EasySep magnet for 5 min. Cells were poured into a new tube and treated for an additional 5 min with 100 µl of magnetic RapidSpheres and placed on the EasySep magnet for 5 min twice more. Cells were then spun down and resuspended in HBSS containing 0.1% HSA without Ca^2+^ and Mg^2+^ and kept at 1 × 10 cells/mL on ice after isolation and prior to experimentation. Cell purity was >90% as determined by Beckman cell coulter counter. Cell viability was ~99% as determined by flow cytometry detection of zombie violet fluorescent dye staining (Biolegend).

### Flow Cytometry Detection of Cell Surface Marker Expression

Neutrophils (1 × 10^6^ cells/ml) in HBSS buffer containing Ca2+ and Mg2+ at 1 mM were treated with IL-8 at a dose range of concentrations (0.01–100 nM) and Feraheme (1–6 mg/ml) for 5 min at 37°C before addition of antibody (1.5–5 µg/ml). Cells were stained with antibodies for high affinity β2-integrin (mAb24), L-Selectin (Dreg-56), CD11b (M1/70), CD18 (1B4), CD11a (HI111), PSGL-1(KPL-1), CXCR1(8F1/CXCR1), and CXCR2 (5E8/CXCR2) for 20 min at 37°C before fixation with 4% paraformaldehyde at room temperature. After two washes with PBS cells were analyzed using the Attune NxT flow cytometer (Thermofisher). Neutrophils were gated by their characteristic forward scatter vs side scatter profile. Receptor expression in terms of sites/cell was determined by comparing the MFI of bound antibody to Quantum Simply Cellular beads (Bangs Laboratories, Inc., Fishers, IN) which contain five bead sets with increasing numbers of antibody binding sites on their surface. From this analysis, a linear relation between MFI and receptor expression was determined for each directly conjugated antibody bound to cells and the calibration bead set.

### Microfluidic Shear Flow Assay

Vascular mimetic flow chambers were utilized to record neutrophil rolling and arrest behavior on substrates of endothelial ligands under physiological shear stresses. Devices were prepared as described previously (27). Briefly, polydimethylsiloxane (PDMS) microfluidic flow chambers with a minimum feature size of 5 μm were produced by curing Sylgard 184 prepolymer (Dow Corning, Midland, MI, USA) over a patterned silicon wafer. Holes were punched into the PDMS for flow channel and vacuum port access and the device was reversibly vacuum sealed on a glass coverslip coated with E-selectin (1 µg/ml) alone or with ICAM-1 (1 µg/ml) in PBS for 60 min at room temperature. To limit non-specific adhesion of neutrophils, coverslips were treated with 1% casein for 10 min before washing with PBS and assembly of the microfluidic device.

Isolated neutrophils were treated with Ca2+ indicators 1 Fluo-4 AM or Rhod-2 AM (1 µg/ml) for 20 min at room temperature in the dark. Cells were spun down and resuspended at 1 × 10^6^ cells/ml and were treated with Mac-1 blocking antibody M1/70 and incubated with or without MNP in HBSS buffer containing Ca2+ and Mg2+ for 20 min at 37°C. Neutrophils were then treated with 0.5 nM IL-8 or vehicle control and immediately loaded into an open 100-µl reservoir and were drawn through the channel by negative pressure produced by a syringe pump at a shear of 2 dynes/cm^2^.

Utilizing real time fluorescence microscopy, images of rolling neutrophils were acquired at 60 frames per minute for 4 min per field of view on an inverted microscope (Nikon) using a phase contrast 20× objective and recorded with 16-bit digital complementary metal oxide semiconductor (CMOS) camera (Andor ZYLA) connected to a PC (Dell) with NIS Elements imaging software (Nikon Instruments Inc.). Arrested neutrophils were defined as having a velocity less than 0.1 µm/s. Migrating neutrophils were identified by exhibition of a polarized shape defined as exceeding a length/width aspect ratio greater than 1.4 and phase dark contrast indicative of being outside the focal plane of rolling cells.

### L-Selectin and PSGL-1 Clustering

Total internal reflection fluorescence (TIRF) microscopy and quantitative dynamic foot printing (qDF) were employed to record fluorescently tagged antibodies targeting L-selectin (non-blocking clone: DREG55) and PSGL-1(non-blocking clone: PL2). Cluster area and frequency during rolling of isolated neutrophils over E-selectin in the presence or absence of Feraheme was observed. A cluster was defined as an area of uniform fluorescence intensity two standard deviations above the mean fluorescence intensity of the cell. Additionally, a cluster was defined as having a surface area of 0.4 µm^2^ or greater.

### Measurement of Reactive Oxygen Species

Neutrophils (1 × 10^6^ cells/ml) in HBSS buffer containing Ca2+ and Mg2+ at 1 mM were incubated with the ROS indicator Dihydrorhodamine 123 (2 µM) and treated with or without IL-8 (1 nM) and Feraheme (4 mg/ml) for 10 min at 37°C before addition of 1 µM fMLP. Cells were incubated with fMLP at 37°C for 5 min and then placed on ice to end reactions and ROS was then quantified through flow cytometry.

### Measurement of Calcium Flux

Isolated neutrophils were treated with the Ca2+ indicator Fluo-4 AM (1 µg/ml) for 20 min at room temperature in the dark. Cells were spun down and resuspended at 1 × 10^6^ cells/mL in HBSS buffer containing Ca2+ and Mg2+ at 1 mM and treated with or without the adenosine A_2A_ receptor antagonist ZM 241385 (2.5 µM) for 5 min at 37C. Feraheme (4 mg/ml) and or adenosine A2a receptor agonist CGS 21680 (1 µM) were then added to the cell solutions for 10 min at 37C. After incubation, IL-8 (1 nM) was added to cell solutions and calcium fluorescence time course was immediately read on the Facscan flow cytometer (BDbiosciences)

## Results

### Feraheme Inhibits Neutrophil Activation and Degranulation Stimulated by IL-8

**Figure 1:**
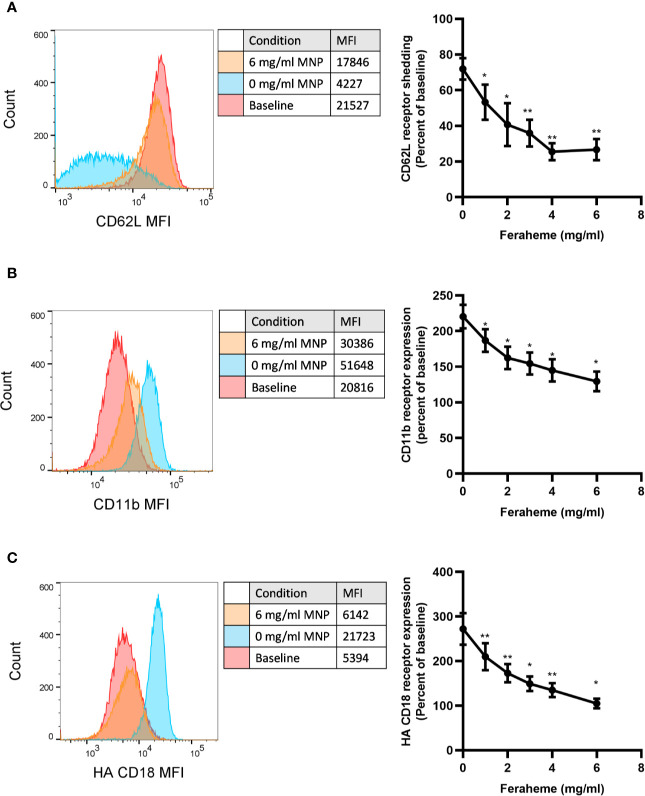
Effect of Feraheme concentration on integrin and selectin expression following chemotactic stimulation of neutrophils. Isolated human neutrophils were incubated with 1 nM IL-8 and Feraheme magnetic nanoparticles (MNP) for 25 min and cell surface expression of **(A)** CD62L, **(B)** CD11b, and **(C)** HA CD18 was assessed by flow cytometry. Representative histograms depict fluorescent antibody detection for each adhesion receptor at baseline receptor expression and following IL-8 stimulation in presence and absence of MNP. Bivariate data are presented as the percent shedding from unstimulated baseline mean ± SEM for CD62L and percent of unstimulated baseline expression mean ± SEM for CD11b and HA CD18 (n ≥ 4 donors) with experimental replicates averaged for each donor. Paired T-test was performed comparing the average value at each concentration to the 0 mg/ml Feraheme condition of the same donor * and ** denote p value ≤.05 and ≤ 0.01, respectively.

Stimulation with chemotactic factors activate within seconds neutrophil degranulation, which results from the fusion of granule membranes to the plasma membrane resulting in upregulation of additional CD11b/CD18 and CD66b receptors. Activation also results in a shift in the conformation of CD18 from a constitutive low affinity conformation at rest to high affinity upon CXCR ligation, as well as the shedding of L-selectin receptors through the action of the metalloprotease ADAM17 (28, 29). We first assessed the capacity of different concentrations of Feraheme in suspension to alter neutrophil responses to chemotactic activation *via* CXCR1/2 in cells stimulated at the K_D_ of IL-8 stimulation (~1 nM). Neutrophils were incubated at 37°C in the presence and absence of Feraheme and IL-8 for 5 min before the addition of fluorescent antibodies for 20 min followed by cell fixation. The presence of Feraheme led to significant alterations in expression of CD62L, CD11b, and high affinity (HA) CD18 at each dose applied (1–6 mg/ml) (**Figure 1**). A dose dependent increase in the inhibition of degranulation was observed, as well as diminished activation of integrin and proteolytic cleavage of L-selectin. Since the effect of Feraheme was observed to plateau at 4 mg/ml, subsequent studies were performed using this concentration.

**Figure 2:**
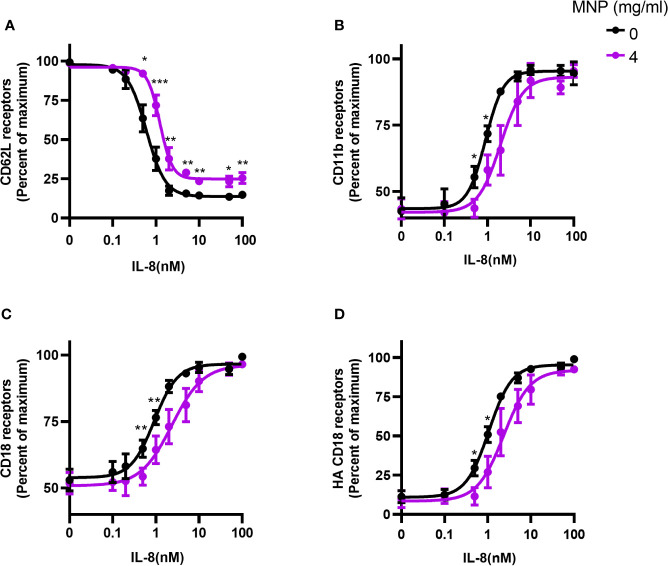
Feraheme alters adhesion receptor expression over a dose range in stimulation with IL-8. Isolated human neutrophils were incubated with IL-8 and Feraheme MNP for 25 min and cell surface expression of **(A)** CD62L, **(B)** CD11b, **(C)** CD18, and **(D)** HA CD18 was assessed by flow cytometry. The data are presented as the percent of maximum receptor expression mean ± SEM (n ≥ 5 donors) with experimental replicates averaged for each donor. Paired T-tests were performed comparing the average of the 0 mg/ml to the 4 mg/ml conditions for the same donor *, **, and *** denote p value ≤.05, ≤ 0.01, and ≤ 0.001, respectively.

We next evaluated the effect of Feraheme on adhesion receptor expression over a dose range of chemotactic stimulation with IL-8. A typical sigmoidal dose dependent decrease in L-selectin expression followed addition of IL-8 between 0.1 and 100 nM, which reached a maximum of ~90% loss at 5 nM IL-8 from baseline expression on quiescent neutrophils (**Figure 2A**). Upregulation in surface expression of CD11b and CD18 provided a sensitive measure of activation, increasing by 2-fold and 2.3-fold from baseline up to maximum expression at 10 nM IL-8 (**Figures 2B, C**). Another sensitive measure of neutrophil activation was provided by the increased binding of mAb24 that reports on the allosteric conversion of CD18 receptors from low to high affinity, which yielded a 3-fold increase in expression at maximum IL-8 stimulation (**Figures 2B, D**). It is noteworthy that the maximum extent of L-selectin shedding was lowered by Feraheme at 4 mg/ml compared with 0 mg/ml, as indicated by the elevated plateau at a dose of IL-8 > 5 nM from 10% up to 25% total receptor expression (**Figure 2A**). Moreover, the extent of upregulation of CD11b/CD18 and conversion of CD18 to the HA conformation was significantly inhibited, as indicated by a ~2-fold increase in the EC50 of IL-8 stimulation. This resulted in a reduced sensitivity to activation that was significant at IL-8 doses of 0.5 and 1 nM for CD11b, total CD18, and HA CD18 (**Figures 2B–D**). A marker of the release of secondary granules is the increase in membrane expression of CD66b, which is transported to the plasma membrane along with CD11b/CD18 (20). The presence of Feraheme elicited a significant inhibition in upregulation of CD66b, increasing the EC50 of IL-8 stimulation by ~30% (**Supplemental Figure 1**). In contrast, CD11a and PSGL-1 adhesion receptors that are constitutively expressed on circulating neutrophils and typically do not register a change in receptor number in response to chemotactic stimulation, remained constant over the dose range of IL-8 stimulation in the presence of Feraheme (**Supplemental Figure 1**).

**Figure 3:**
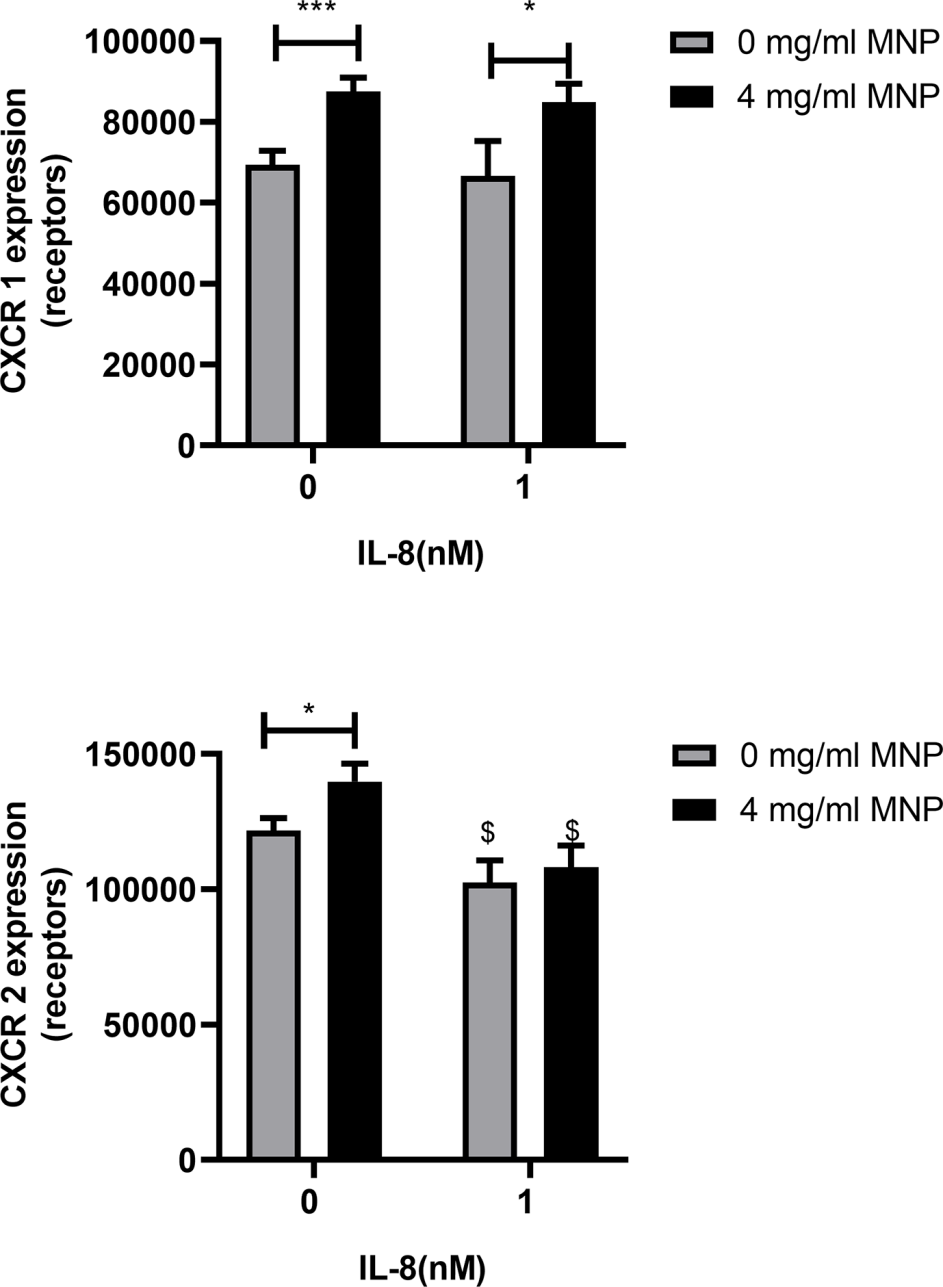
Feraheme alters CXCR expression in neutrophils. Isolated human neutrophils were incubated with IL-8 or vehicle and Feraheme for 25 min and cell surface expression of **(A)** CXCR1 and **(B)** CXCR2 was assessed by flow cytometry. The data are presented as mean ± SEM (n ≥4 donors) with experimental duplicates averaged for each donor. Paired T-tests were performed comparing the average value for the 4 mg/ml to the 0 mg/ml Feraheme conditions of the same donor * and *** denote p value ≤.05 and ≤ 0.001, respectively. Paired T-tests were performed comparing the average value for a condition to the 0 nM IL-8 condition of the same donor $ denotes p value ≤.05.

Neutrophils are reported to endocytose CXCR1/2 following ligation and signaling by IL-8 (30). This motivated experiments to determine if Feraheme altered the expression of CXCR1/2, consequently accounting for the diminished capacity to induce changes in adhesion molecule expression following stimulation. Unexpectedly, incubating unstimulated neutrophils in suspension with Feraheme at 4 mg/ml elicited a ~26% increase in expression of CXCR1 and a ~15% increase in CXCR2 (**Figure 3**). A significant amount of CXCR1 endocytosis following IL-8 simulation was not observed in the presence or absence of Feraheme (**Figure 3A**). In contrast, the small amount of CXCR2 endocytosis in response to IL-8 stimulation was not altered in the presence of Feraheme (**Figure 3B**). These results indicate that CXCR1/2 expression either remains constant or increases in the presence of Feraheme and IL-8 stimulation as compared to controls. Thus, it is unlikely that the mechanism left-shifting the IL-8 dose response for activation is diminished expression of chemotactic receptors. Feraheme is also not altering the endocytosis of CXCR2 receptors in cells treated with 1 nM IL-8 indicating it is not interfering with IL-8 ligation of CXCR2. Thus, it is probable that Feraheme is leading to dramatically reduced responses to IL-8 stimulation by affecting intracellular signaling downstream of receptor ligation.

### Feraheme Inhibits the Activation of ROS Production in Neutrophils

**Figure 4:**
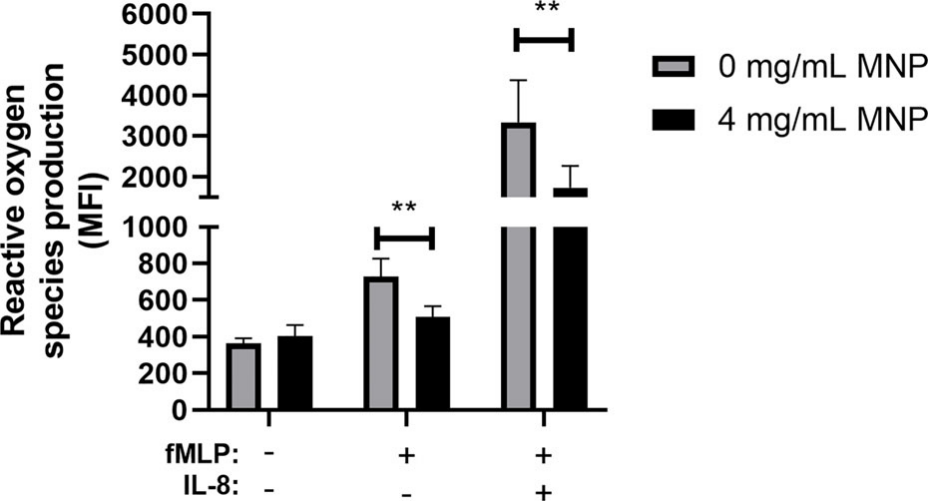
Feraheme inhibits ROS production in fMLP stimulated neutrophils. Isolated human neutrophils stained with 2 µM DHR 123 and treated with or without Feraheme were preincubated with 1 nM IL-8 or vehicle control for 10 min before addition of 1 µM fMLP or vehicle control for 5 min. The data are presented as mean ± SEM (n ≥ 4 donors) with experimental duplicates averaged for each donor. Paired ratio T-test was performed comparing the average value for an experimental condition to the 0 mg/ml Feraheme condition of the same donor. ** denotes a p value ≤ 0.01.

To further explore the influence of Feraheme on neutrophil function, ROS production was quantified using flow cytometry to detect intracellular Dihydrorhodamine 123 fluorescence. Production of ROS is a key antimicrobial mechanism of neutrophils and has also been implicated in recruitment to sites of inflammation by increasing vascular permeability (31). The bacterial tripeptide fMLP is a potent chemotactic factor that binds to G-protein coupled Formyl peptide receptors expressed on neutrophils and activates the respiratory burst and production of ROS. Feraheme led to a significant 30% reduction of ROS production in neutrophils stimulated with 1 µM fMLP (**Figure 4**). Pre-treatment with IL-8 has previously been reported to prime neutrophils for enhanced fMLP induced ROS production through assembly of the NADPH oxidase components into lipid rafts on the plasma membrane (32). Feraheme significantly reduced ROS production by 48% in cells primed with low dose IL-8 and stimulated with fMLP. While uptake of SPION other than Feraheme have been reported to induce ROS production, the presence of Feraheme did not lead to detectable ROS production in unstimulated neutrophils. This data indicates that Feraheme inhibits formyl peptide receptor signaling of ROS production, including increased ROS induced by IL-8 priming.

### Neutrophil Recruitment on Endothelial Adhesion Molecules Is Altered in the Presence of Feraheme

**Figure 5:**
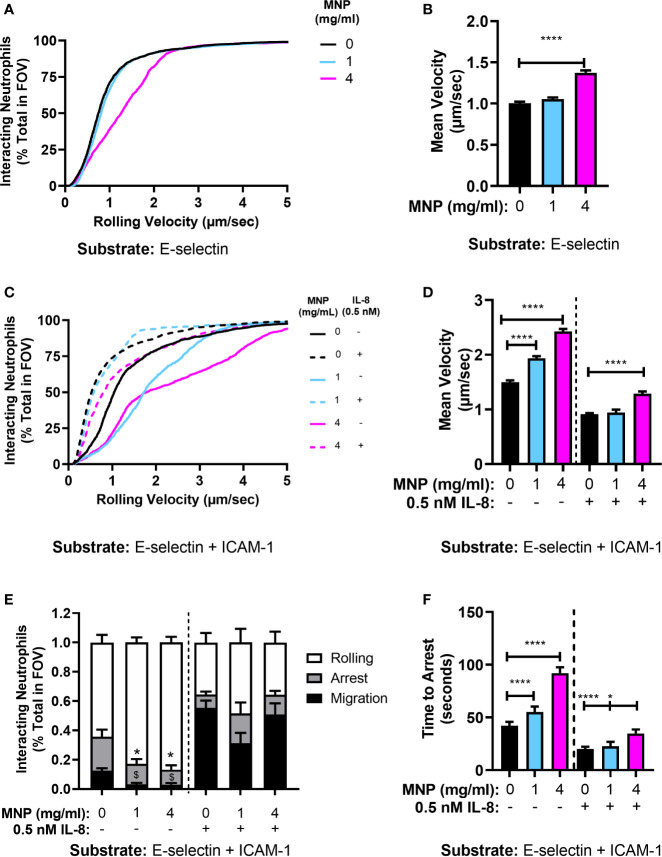
Feraheme alters the kinetics of neutrophil rolling and arrest in microfluidic flow channels. **(A)** Neutrophil rolling velocity over an E-selectin substrate treated with vehicle or Feraheme (1 mg/ml, 4 mg/ml). **(B)** Cumulative rolling velocities of neutrophils over an E-selectin substrate treated with vehicle or Feraheme (1 mg/ml, 4 mg/ml). **(C)** Mean neutrophil rolling velocity over an E-selectin+ ICAM-1 substrate treated with Feraheme (1mg/ml, 4 mg/ml) and/or IL-8 (0.5 nM). **(D)** Cumulative rolling velocities of neutrophils over an E-selectin + ICAM-1 substrate treated with vehicle or Feraheme (1 mg/ml, 4 mg/ml) and/or IL-8 (0.5 nM). **(E)** Neutrophil rolling to arrest and migration over an E-selectin+ ICAM-1 substrate treated with Feraheme (1 mg/ml, 4 mg/ml) and/or IL-8 (0.5 nM). * and ^$^ denote T-Test p-values of ≤.05 as compared to the 0 mg/ml MNP condition for arrest and migration, respectively. **(F)** Neutrophil time to arrest over an E-selectin+ ICAM-1 substrate treated with Feraheme (1 mg/ml, 4 mg/ml) and/or IL-8 (0.5 nM). For B, D, and F, * and **** denote T-Test p-values of ≤.05 and ≤ 0.0001, respectively. Data in B, D, E, and F are presented in mean ± SEM (n ≥ 4 donors) with at least 15 cells for each donor per condition.

It has been reported that exposure to nanoparticles and microparticles reduced neutrophil recruitment to inflamed mesentery microvasculature in mice (23). Moreover, polystyrene nanoparticles have been shown to reduce adhesion of human neutrophils to inflamed human umbilical vein endothelial cells in parallel plate flow chamber studies (24). This motivated determination of the effect of Feraheme on the multistep process of recruitment *in vitro* using microfluidic flow channels (23, 24, 33). Fluorescence microscopy was employed to image the dynamics of neutrophil rolling and arrest within vascular mimetic microfluidic channels containing substrates coated with E-selectin alone or in conjunction with ICAM-1. Neutrophil suspensions were incubated for 20 min with Feraheme at 1 mg/ml or 4 mg/ml and compared with vehicle control in response to stimulation with 0.5 nM IL-8 prior to perfusion through the flow channel. This dose of IL-8 corresponded with the lowest concentration of stimulus in which a significant inhibition of CD18 activation and L-selectin shedding was elicited by Feraheme. Capture and rolling on E-selectin is primarily mediated by recognition of sLe^x^ presenting ligands on L-selectin and PSGL-1 expressed on neutrophils. The velocity of neutrophil rolling on E-selectin at a venular shear stress of 2 dyne/cm^2^ increased ~37% in the presence of Feraheme at 4 mg/ml compared to vehicle alone, whereas no alteration in rolling velocity was detected at 1 mg/ml (**Figures 5A, B**). This increased rolling velocity was accompanied by a 40% increase in the variance of the mean velocity, which is consistent with interruption of adhesive bond formation resulting in an unsteady trajectory of neutrophil rolling on the substrate under shear flow. Neutrophils require ICAM-1 on the substrate to provide an anchor for activated integrins to bind in order to achieve shear resistant cell arrest (28). Perfusion through flow channels derivatized with E-selectin and ICAM-1 were used to study Feraheme’s effect on the kinematics of neutrophil arrest and transition to a migratory phenotype. In the absence of IL-8 chemotactic stimulation, Feraheme lead to a dose dependent increase in rolling velocity on E-selectin coated substrates, which increased by ~30% and ~60% when neutrophils were exposed to 1 and 4 mg/ml Feraheme, respectively (**Figures 5C, D**). Moreover, an ~80% increase in variance of mean velocity corresponded to treatment at 4 mg/ml Feraheme, while 1 mg/ml increased variance by 17%. Stimulation with IL-8 leads to the activation of HA CD18 that binds ICAM-1 and mediates deceleration to arrest (28). Consequently, stimulation with IL-8 on E-selectin/ICAM-1 coated substrates resulted in a 50% decrease in rolling velocity and within ~40 s most neutrophils exhibited a rapid transition to arrest (**Figures 5C, F**). Neutrophils treated with Feraheme at 4 mg/ml exhibited ~40% higher rolling velocity compared to the 1 mg/ml or vehicle control conditions (**Figure 5D**). In the absence of IL-8 stimulation, outside-in signaling *via* E-selectin binding and clustering of L-selectin is sufficient to activate ~40% of cells to convert from rolling to arrest that is supported by activation of HA CD18 to bind ICAM-1 (34). Feraheme at 1 and 4 mg/ml concentrations inhibited this L-selectin mediated signaling of arrest, as well as decreased transition to a migratory state from 12% of cells to <1% of cells assuming a polarized shape **(** **Figure 5E**). In contrast, in the presence of IL-8 stimulation, we did not detect significant inhibition in the frequency of neutrophils rolling to arrest in presence of Feraheme (**Figure 5E**). This is likely due to the fact that coupled with shear force mediated L-selectin signaling, even very low concentrations of IL-8 can lead to activation of HA CD18 receptors sufficient to mediate conversion from rolling to arrest (35). However, in the presence of Feraheme, a significant increase was detected in the duration of rolling before the onset of cell arrest for both IL-8 stimulated and unstimulated neutrophil suspensions. In the absence of IL-8, rolling time to arrest increased from 42.1 seconds in the 0 mg/ml condition to 55.1 and 91.9 seconds in the 1 mg/ml and 4 mg/ml conditions, respectively (**Figure 5F**). In IL-8 treated cells only the 4 mg/ml condition resulted in significantly increasing the time to arrest compared to the 0 mg/ml condition, from 20.0 to 34.7 s. We conclude that Feraheme exerts a significant effect on the kinematics of rolling to arrest on E-selectin and ICAM-1 in terms of increasing the velocity and signaling associated with HA CD18 mediated arrest.

### Feraheme Perturbs the Process of E-Selectin Ligand Clustering on Rolling Neutrophils

**Figure 6:**
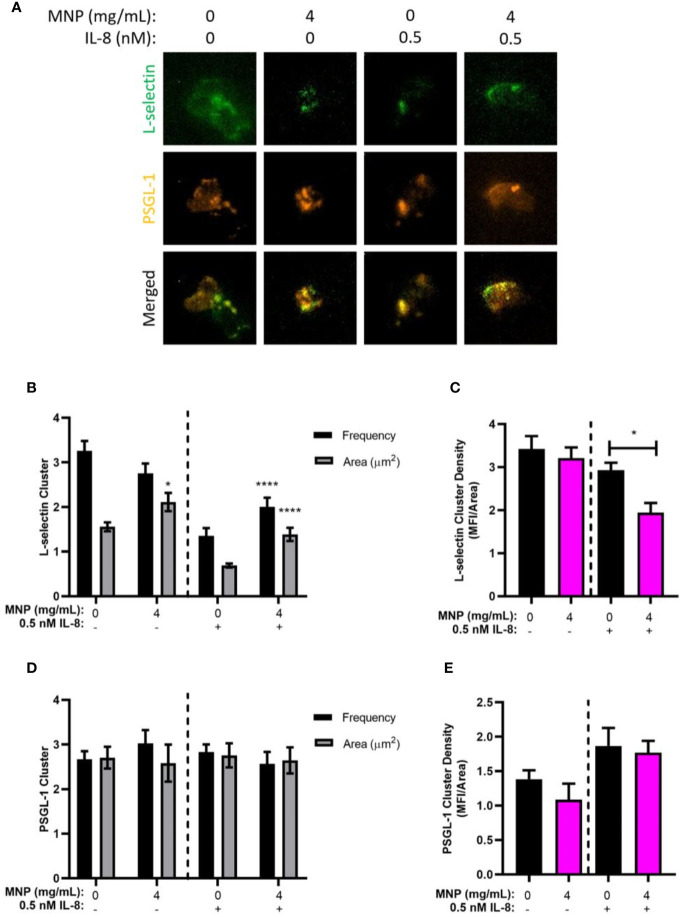
Feraheme antagonism of E-selectin ligand bond formation. **(A)** Neutrophil rolling on a substrate of E-selectin in the presence of vehicle control or Feraheme MNP (4 mg/ml) was dynamically imaged using qDF to detect L-selectin (AF488 anti-human DREG55) and PSGL-1 (PE anti-human PL-1) engagement in the plane of adhesive contact. **(B)** L-selectin and **(C)** PSGL-1 receptor cluster area and frequency were determined. **(D)** L-selectin and **(E)** PSGL-1 receptor density was determined and reported as mean ± SEM (n = 3 donors) with at least 10 cells for each donor per condition. * and **** denote T-test p-values of ≤.05 and ≤ 0.0001, respectively, compared with the 0 mg/ml condition.

E-selectin recognition of L-selectin and PSGL-1 under shear flow leads to receptor co-clustering and mechanotransduction of signals leading to HA CD18 binding to ICAM-1 (34). To further examine how uptake of Feraheme effects the process of selectin mediated rolling and deceleration to arrest, total internal reflection fluorescence (TIRF) microscopy and quantitative dynamic footprinting (qDF) was performed to determine the L-selectin and PSGL-1 bond cluster formation during rolling on a substrate of E-selectin. Treatment with Feraheme resulted in an increase in L-selectin bond cluster area (i.e., lower density of L-selectin) and marginal changes in the frequency of cluster formation detected in the presence and absence of IL-8 stimulation (**Figures 6A, B**). Examining the average density of L-selectin receptors within clusters bound to E-selectin, a significant decrease was detected in the presence of IL-8 stimulation for 4 mg/ml compared to 0 mg/ml Feraheme (**Figure 6C**). In contrast, Feraheme did not affect E-selectin mediated formation of PSGL-1 cluster area and frequency nor PSGL-1 cluster density (**Figures 6D, E**). These data are consistent with the observed increase in rolling velocity and decreased outside-in signaling *via* E-selectin in the presence of Feraheme and indicate this effect may be a function of diminished L-selectin bond formation despite the observed higher frequency and area of L-selectin within sites of adhesive contact.

### Feraheme Accelerates Clearance of Cytosolic Calcium After Flux

**Figure 7:**
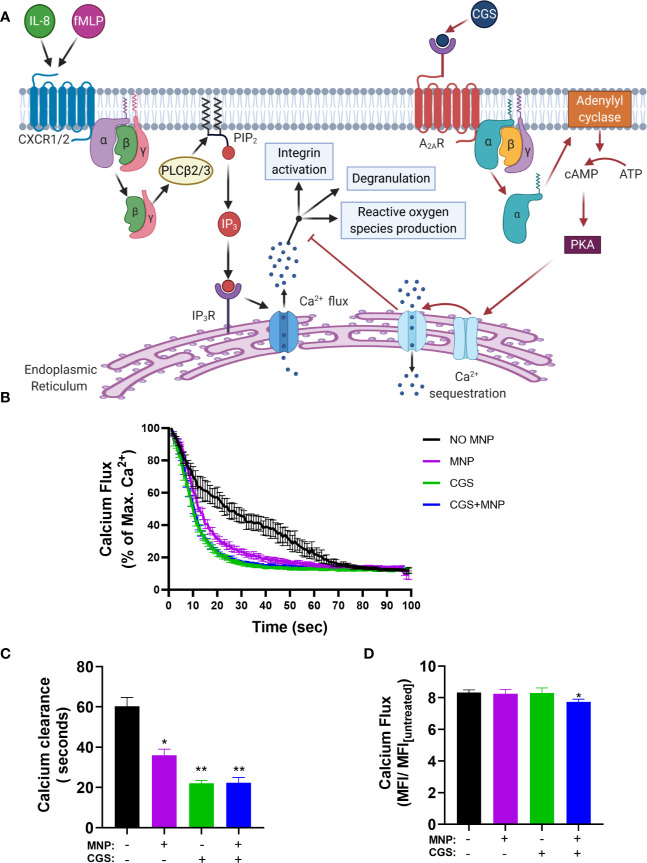
Feraheme (4 mg/ml) accelerates clearance of cytosolic calcium following IL-8 stimulated calcium flux. **(A)** Diagram depicts the pathway of Ca2+ mediated neutrophil activation and antagonism by CGS 21680 via the Adenosine A_2A_ receptor. **(B)** Kinetics of cytosolic calcium clearance following maximal release due to 1 nM IL-8 at t = 0 s. **(C)** Duration for Ca2+ clearance to reach 20% of maximum value. **(D)** Fold change between maximal calcium flux and baseline level in untreated cells. Data are presented in mean ± SEM (n = 3 donors) with experimental replicates for each donor. Paired T-tests were performed comparing experimental conditions to the NO MNP condition of the same donor *, and ** denote p value ≤.05, and ≤ 0.01, respectively.

Calcium serves as a secondary messenger downstream of GPCR signaling that mediate neutrophil inflammatory responses including degranulation, integrin activation and adhesion, shape change, and ROS production (36). IL-8 ligation of CXCR leads to a release of endoplasmic reticulum (ER) stored calcium into the cytosolic space which is subsequently sequestered by the ER calciosomes to replenish stores. High concentrations of extracellular adenosine which binds to the adenosine A_2A_ receptor is known to dampen neutrophil inflammatory responses (37). The mechanism, as depicted in **Figure 7A**, is initiated by ligand binding to the adenosine A_2A_ receptor which induces disassociation and activation of the G_α_ subunit of the A_2A_ receptor linked heterotrimeric G protein which then activates adenylyl cyclase. Adenylyl cyclase activation leads to production of cyclic AMP (cAMP), which in turn activates cAMP-dependent protein kinase (PKA) and leads to accelerated sequestration of calcium through PKA-activated endo-membrane Ca^2+^-ATPases. To determine if Feraheme’s inhibitory effects on intracellular signaling are mediated through accelerated clearance of IL-8 induced calcium flux, the time course of cytosolic calcium stimulated by IL-8 in Feraheme treated neutrophils was compared in the presence and absence of adenosine A_2A_ receptor agonist CGS 21680 (CGS). Following IL-8 induced release of ER Ca^2+^, Feraheme elicited an accelerated decrease in cytosolic calcium as compared to untreated cells. The level of accelerated Ca^2+^ clearance was equivalent and not additive with CGS (**Figure 7B**). The duration required for a decrease to 20% of the maximum Ca2+ flux elicited by IL-8 was 60 seconds in untreated cells compared to 36, 22, and 22 seconds for Feraheme, CGS, and Feraheme + CGS, respectively (**Figure 7C**). Neither Feraheme nor CGS alone reduced the maximal Ca^2+^ flux elicited by IL-8, while added together they resulted in a slight reduction (**Figure 7D**).

**Figure 8:**
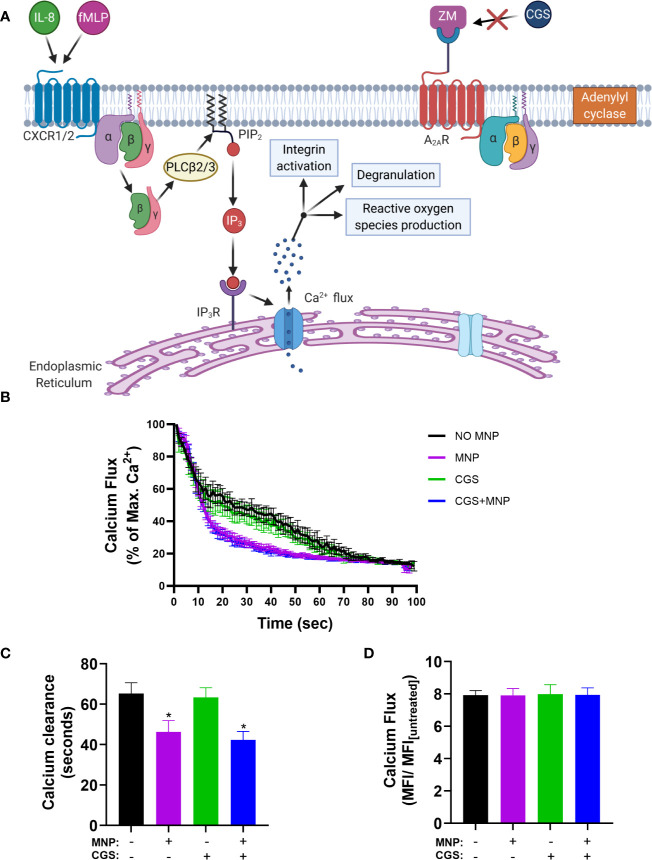
Feraheme MNP (4 mg/ml) accelerated clearance of IL-8 stimulated cytosolic Ca2+ is independent of the Adenosine A_2A_ receptor. The Adenosine A_2A_ receptor antagonist ZM (2.5 µM) was applied for 5 min in calcium buffer. **(A)** Diagram depicts calcium flux in cells preincubated with ZM before treatment with the Adenosine A_2A_ receptor agonist CGS **(B)** Kinetics of cytosolic calcium resequestration from maximal flux induced by IL-8 (1 nM) at t=0 sec. **(C)** Time from maximal calcium flux until calcium levels reach 20 percent of max value. **(D)** Fold change between maximal calcium flux and untreated cells. Data are presented in mean ± SEM (n=3 donors) with experimental replicates for each donor. Paired T-tests were performed comparing experimental conditions to the NO MNP condition of the same donor. * denotes a p value ≤.05.

To determine if Feraheme mediated inhibition acts through release and binding of extracellular adenosine, the clearance of calcium after IL-8 stimulated Ca^2+^ flux was compared to that activated by the adenosine A_2A_ receptor agonist CGS 21680 (CGS) while blocking signaling *via* the A_2A_ receptor with the adenosine A_2A_ receptor antagonist ZM 241385 (ZM) as depicted in **Figure 8A**. As expected, in neutrophils preincubated with ZM the sequestration effect of CGS was completely abrogated. In contrast, Feraheme treatment remained effective in accelerating the decrease in cytosolic Ca^2+^ following IL-8 stimulation (**Figure 8B**). In untreated cells the duration to 20% of maximum Ca^2+^ flux was 65 s compared to 46, 63, and 42 s for Feraheme, CGS, and Feraheme+ CGS, respectively (**Figure 8C**). All conditions registered the same maximal Ca^2+^ flux (**Figure 8D**).

## Discussion

Neutrophil recruitment and attachment to inflamed endothelium is mediated by a multistep process involving ligation and mechanosignaling from the outside-in through selectins and integrins following bond formation (38). CXCR ligation of IL-8 by neutrophils interacting with inflamed endothelium under shear flow induces degranulation and the activation of CD18 to a high affinity state, as well as the rapid shedding of L-selectin (20, 29). Here, we demonstrated that, treating neutrophils in suspension over a dose range with Feraheme elicited reduced responsiveness to stimulation with 1 nM IL-8 at each dose tested (1–6 mg/ml). This was manifest as increased inhibition of CD11b upregulation, HA CD18 activation, and L-selectin shedding with increasing particle concentration of Feraheme. Treating with a Feraheme dose of 4 mg/ml resulted in a consistent ~2-fold right shift in the EC50 of stimulation with IL-8 corresponding to diminished upregulation of CD11b/CD18, activation of HA CD18, and L-selectin shedding. Chemokine receptor expression in unstimulated cells was significantly elevated in the presence of Feraheme, but these changes in expression did not explain the inhibitory effects observed. Feraheme also did not alter endocytosis of CXCR2 in response to IL-8 signaling, suggesting that its mechanism of action is not through down regulation or interference with CXCR ligation. Feraheme also significantly reduced fMLP induced ROS production in the presence or absence of IL-8 priming. Taken together, we conclude that the inhibitory effects of Feraheme are not exclusive to CXCR1/2 signaling by IL-8, but include signaling through other GPCR namely, formyl peptide receptors.

Uptake of nanoscale poly(styrene) and liposomal particles by neutrophils was previously shown not to enhance apoptosis, activation, or cell death. Ingested nanoparticles were reported to reside in intracellular compartments that are retained during degranulation (39). Uncoated SPION are reported to aggregate and induce the formation of neutrophil extracellular traps (NETs) that are extracellular fibers consisting of expelled DNA. This pro-inflammatory host response is mitigated by coating SPION with layers of dextran or HSA (40). In our experiments, Feraheme did not reduce cell viability, which exceeded 99%. Although we did not specifically test for netosis in our studies of neutrophil arrest and migration, no visible changes in cell morphology that is characteristic of netosis was observed. In-vivo studies have demonstrated that uptake of nanoparticles can diminish the efficiency of neutrophil recruitment to inflamed endothelium within the lungs of mice. However, the mechanism that may involve cell-particle uptake and competitive binding to adhesion receptors, which could interfere with chemotactic or adhesive ligand binding during recruitment remained elusive (23, 24). Here, we report that following 20 min of exposure to Feraheme in suspension, we observed diminished efficiency of neutrophil arrest and migration as measured in an established model of endothelial inflammation in vascular mimetic shear flow channels. We have recently reported that deceleration of neutrophils within the vascular mimetic substrate in microfluidic flow channels is mediated by as few as 200 HA CD18 binding ICAM-1 in order to transition from rolling to arrest (34). In the absence of IL-8 stimulation and in the presence of Feraheme, a ~50% reduction was measured in the frequency of neutrophils rolling to arrest on E-selectin/ICAM-1 at both 1 and 4 mg/ml. Moreover, neutrophils rolled over twice the distance before activation and binding of HA CD18 integrin bonds formed stable arrest. Inhibition of neutrophil CD11b/CD18 mediated migration after arrest was also observed at 1 and 4 mg/ml Feraheme. Thus, Feraheme uptake in a concentration dependent manner alters both selectin and GPCR mediated signaling of HA CD18 necessary to transition from rolling on E-selectin to arrest on ICAM-1. It has been previously shown that L-selectin catch-bonds, characterized by a prolonged bond lifetime as tensile force is increased, is necessary and sufficient to signal CD18 transition to a HA state (34). We show that Feraheme reduced both L-selectin shedding in response to IL-8 and L-selectin clustering during rolling on E-selectin, but it had no effect on PSGL-1 expression. Noteworthy was the finding that cluster area of L-selectin bonds during rolling on E-selectin in the presence of shear stress and IL-8 stimulation was increased, while the density of bond formation within these clusters decreased. This indicates that Feraheme may alter L-selectin signaling, but not recognition by E-selectin on the surface of rolling neutrophils. This is consistent with the significant rise in the mean rolling velocity on E-selectin that was observed to increase with Feraheme concentration. This provides one potential explanation as to how Feraheme reduces the efficiency of the transition to cell arrest, in that it may alter the capacity of E-selectin/L-selectin to form catch-bonds. Increased availability of L-selectin to form bond clusters in the presence of Feraheme coincided with reduced density of bonds on E-selectin and also with a reduced frequency of cell arrest suggesting that the normal process of neutrophil deceleration at sites of inflammation may be perturbed. Reduced levels of L-selectin shedding may potentially contribute to the inhibition of arrest and polarization observed in Feraheme treated cells. Consistent with the latter observation are reports that L-selectin shedding has been reported to amplify integrin-mediated outside-in signaling–dependent processes, including neutrophil migration, production of ROS, and phagocytosis (41). L-selectin shedding induces phosphorylation of phospholipase C (PLC)γ2, Akt, and Syk, critical regulators of intracellular calcium release that are important for E-selectin mediated slow rolling and CD18 integrin activation in neutrophils (41, 42). Thus, inhibition of L-selectin shedding may also interfere with normal activation of HA CD18 during rolling on E-selectin, despite the elevated expression of L-selectin on the surface. While it is likely that Feraheme is endocytosed by neutrophils in suspension, we cannot rule out that a fraction remain bound to the plasma membrane and sterically interfere with the recognition and mechanics of selectin bond formation. Due to optical limitations in real-time imaging of SPION bound to the plasma membrane at the nanoscale, we could not directly image whether Feraheme specifically bound to L-selectin receptors and sterically interfered with catch-bond formation associated with mechanotransduction of CD18 activation. What is clear is that Feraheme altered the efficiency of L-selectin’s capacity to mechanosignal activation of HA CD18, but not necessarily its ability to bind E-selectin and form focal clusters.

**Figure 9:**
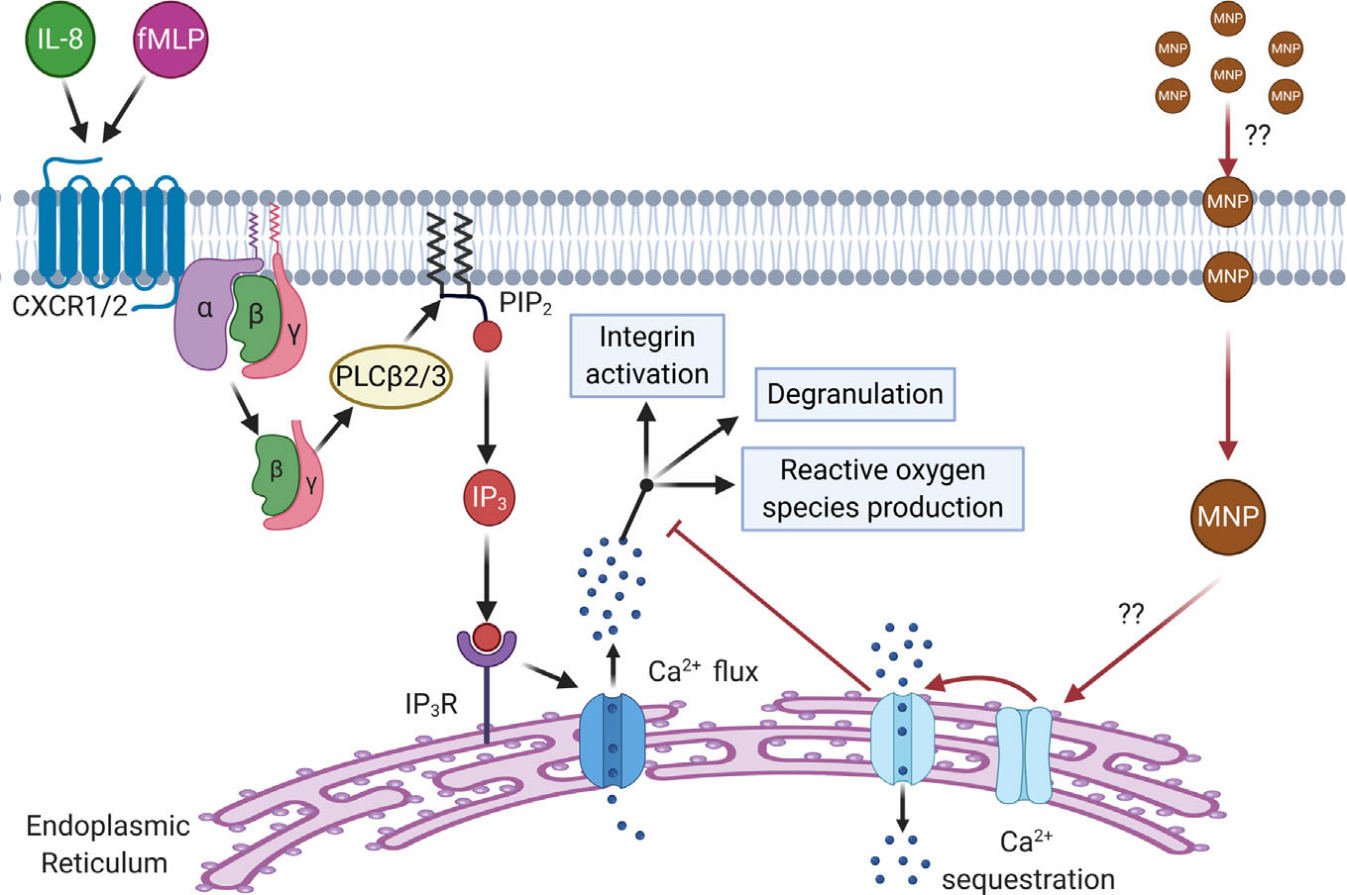
Schematic depicting the proposed mechanism of Ca2+ sequestration and inhibition of downstream response by Feraheme. Feraheme may inhibit neutrophil function through activation of endo-membrane Ca2+-ATPases leading to accelerated clearance of cytosolic calcium inhibiting intracellular signaling. CXCR1/2 ligation by endogenous receptor ligands (IL-8, fMLP) leads to dissociation of Gα from Gβγ subunits of G proteins activating PLCβ2/3 which splits phosphatidylinositol 4,5 biphosphate (PIP_2_). PIP_2_ splits releasing inositol-1,4,5 triphosphate (IP_3_) that binds to IP_3_ receptor (IP_3_R) on the surface of the endoplasmic reticulum inducing calcium flux which signals for downstream effector functions (integrin activation, degranulation, and reactive oxygen species production). We propose that Feraheme MNP are being endocytosed by neutrophils which through an unknown intermediary lead to activation of endo-membrane Ca2+-ATPases that sequester calcium and inhibit calcium signaling of functional responses down stream of GPCR.

To elucidate a possible mechanism for Feraheme inhibition of GPCR signaling the effects of Feraheme on calcium flux after IL-8 stimulation was recorded. Feraheme significantly accelerated the clearance of calcium after flux and this response was comparable to that of the adenosine A_2A_ receptor agonist CGS 21680 known to inhibit degranulation and ROS production. The effect of Feraheme was not dependent on adenosine as treatment with the adenosine A_2A_ receptor antagonist ZM 241385 did not perturb the acceleration of calcium clearance in response to Feraheme. Calcium signaling plays a key role in GPCR mediated degranulation, L-selectin shedding, integrin activation, and ROS production which are all inhibited by Feraheme. Calcium is also necessary for selectin mechanosignaling induced activation of HA LFA-1 and for integrin signaling of shape change and polarization after arrest (38). Accelerated clearance of calcium could potentially result in the decreased arrest and polarization observed in Feraheme treated cells by inhibiting selectin mechanosignaling. These experiments highlight a consistent effect of Feraheme uptake on neutrophil intracellular signaling, but the mechanism which may involve Feraheme steric influence on normal cell surface receptor affinity or avidity in binding ligand needs to be confirmed in future studies. The non-additive inhibition in the presence of Feraheme and CGS could indicate a shared downstream mechanism for the accelerated clearance of Ca^2+^. We propose that Feraheme may induce sequestration of calcium through activation of endo-membrane Ca^2+^-ATPases rather than by efflux of calcium through activation of plasma membrane Ca^2+^-pump, the proposed mechanism of action for CGS (**Figure 9**).

In summary, Feraheme exerted an immunosuppressive effect on neutrophil activation in response to IL-8 and fMLP, as well as outside-in signaling *via* E-selectin during neutrophil recruitment in shear flow. A potential mechanism may involve the observed Feraheme induced acceleration of calcium clearance resulting in a reduced capacity for cell signaling of immune responses. Further studies are warranted to elucidate the mechanism by which Feraheme induces accelerated calcium clearance, potentially though elevation of cAMP leading to downstream activation of endo-membrane Ca^2+-^ATPases, and whether this is the primary mechanism for the inhibition of neutrophil inflammatory responses observed herein. Whether these effects are seen with other SPION formulations is also a relevant question to pursue. These immunosuppressive effects could lead to impaired immune responses to infection or sterile tissue insult in patients treated with Feraheme. The current studies also point to the expanded use of SPION to downregulate the innate immune response as a therapy for ameliorating chronic inflammation and/or autoimmune diseases.

## Data Availability Statement

The raw data supporting the conclusions of this article will be made available by the authors, without undue reservation.

## Ethics Statement

The studies involving human participants were reviewed and approved by UC Davis Institutional Review Board. The patients/participants provided their written informed consent to participate in this study.

## Author Contributions

GG designed the study and performed flow cytometry experiments. VM performed microfluidic experiments. GG and VM performed data analysis. GG wrote initial draft with aid of VM and SS. All authors contributed to the article and approved the submitted version.

## Funding

This work was funded by the following grants from the National Institute of Health (NIH RO1 AI047294 to SS, NIH R01 NR015674 to MK, and NIH T32 HL086350).

## Conflict of Interest

The authors declare that the research was conducted in the absence of any commercial or financial relationships that could be construed as a potential conflict of interest.

## References

[1] Dadfar SM Roemhild K Drude NI von Stillfried S Knuchel R Kiessling F Iron oxide nanoparticles: Diagnostic, therapeutic and theranostic applications. Adv Drug Deliver Rev (2019) 138:302–25. 10.1016/j.addr.2019.01.005 PMC7115878 30639256

[2] Valdiglesias V Fernández-Bertólez N Kiliç G Costa C Costa S Fraga S Are iron oxide nanoparticles safe? Current knowledge and future perspectives. J Trace Elem Med Biol (2016) 38:53–63. 10.1016/j.jtemb.2016.03.017 27056797

[3] Gustafson HH Holt-Casper D Grainger DW Ghandehari H Grainger D Nanoparticle Uptake: The Phagocyte Problem. Nano Today (2015) 10:487–510. 10.1016/j.nantod.2015.06.006 26640510 PMC4666556

[4] Owens DEIII Peppas NA Opsonization, biodistribution, and pharmacokinetics of polymeric nanoparticles. Int J Pharm (2006) 307:93–102. 10.1016/j.ijpharm.2005.10.010 16303268

[5] Barbero F Russo L Vitali M Piella J Salvo I Borrajo ML Formation of the Protein Corona: The Interface between Nanoparticles and the Immune System. Semin Immunol (2017) 34:52–60. 10.1016/j.smim.2017.10.001 29066063

[6] Szeto GL Lavik EB Materials Design at the Interface of Nanoparticles and Innate Immunity. J Mater Chem B (2016) 4:1610–8. 10.1039/C5TB01825K PMC4950994 27453783

[7] Bobo D Robinson KJ Islam J Thurecht KJ Corrie SR Nanoparticle-Based Medicines: A Review of FDA-Approved Materials and Clinical Trials to Date. Pharm Res (2016) 33:2373–87. 10.1007/s11095-016-1958-5 27299311

[8] Wysowski DK Swartz L Borders-Hemphill BV Goulding MR Dormitzer C Use of parenteral iron products and serious anaphylactic-type reactions. Am J Hematol (2010) 85:650–4. 10.1002/ajh.21794 20661919

[9] Wu X Tan Y Mao H Zhang M Toxic Effects of Iron Oxide Nanoparticles on Human Umbilical Vein Endothelial Cells. Int J Nanomed (2010) 5:385–99. 10.2147/IJN.S10458 PMC2950396 20957160

[10] Gaharwar U,RP Iron Oxide Nanoparticles Induced Oxidative Damage in Peripheral Blood Cells of Rat. J Biomed Sci Eng (2015) 8:274–86. 10.4236/jbise.2015.84026

[11] Soenen SJH Himmelreich U Nuytten N De Cuyper M Cytotoxic effects of iron oxide nanoparticles and implications for safety in cell labelling. Biomaterials (2011) 32:195–205. 10.1016/j.biomaterials.2010.08.075 20863560

[12] Jones SW Roberts RA Robbins GR Perry JL Kai MP Chen K Nanoparticle Clearance Is Governed by Th1/Th2 Immunity and Strain Background. J Clin Invest (2013) 123:3061–73. 10.1172/JCI66895 PMC3696555 23778144

[13] Naumenko V Nikitin A Garanina A Melnikov P Vodopyanov S Kapitanova K Neutrophil-mediated transport is crucial for delivery of short-circulating magnetic nanoparticles to tumors. Acta Biomaterialia (2020) 104:176–87. 10.1016/j.actbio.2020.01.011 31945505

[14] Shah A Dobrovolskaia MA Immunological effects of iron oxide nanoparticles and iron-based complex drug formulations: Therapeutic benefits, toxicity, mechanistic insights, and translational considerations Nanomedicine: Nanotechnology. Biol Med (2018) 14:977–90. 10.1016/j.nano.2018.01.014 PMC5899012 29409836

[15] Amulic B Cazalet C Hayes GL Metzler KD Zychlinsky A Neutrophil function: from mechanisms to disease. Annu Rev Immunol (2012) 30:459–89. 10.1146/annurev-immunol-020711-074942 22224774

[16] Etzioni A Frydman M Pollack S Avidor I Phillips ML Paulson JC Brief report: recurrent severe infections caused by a novel leukocyte adhesion deficiency. N Engl J Med (1992) 1327:1789–92. 10.1056/NEJM199212173272505 1279426

[17] Del Fresno C Saz-Leal P Enamorado M Wculek SK Martínez-Cano S Blanco-Menéndez N DNGR-1 in dendritic cells limits tissue damage by dampening neutrophil recruitment. Science (2018) 362:351–6. 10.1126/science.aan8423 30337411

[18] Babin K Antoine F Goncalves DM Girard D TiO2, CeO2 and ZnO nanoparticles and modulation of the degranulation process in human neutrophils. Toxicol Lett (2013) 221:57–63. 10.1016/j.toxlet.2013.05.010 23726862

[19] Couto D Freitas M Vilas-Boas V Dias I Porto G Lopez-Quintela MA Interaction of polyacrylic acid coated and non-coated iron oxide nanoparticles with human neutrophils. Toxicol Lett (2014) 225:57–65. 10.1016/j.toxlet.2013.11.020 24291037

[20] Lacy P Mechanisms of Degranulation in Neutrophils. All Asth Clin Immun (2006) 2:98. 10.1186/1710-1492-2-3-98 PMC2876182 20525154

[21] Winterbourn CC Kettle AJ Hampton MB Reactive Oxygen Species and Neutrophil Function. Annu Rev Biochem (2016) 85:765–92. 10.1146/annurev-biochem-060815-014442 27050287

[22] Noël C Simard JC Girard D Gold nanoparticles induce apoptosis, endoplasmic reticulum stress events and cleavage of cytoskeletal proteins in human neutrophils. Toxicol In Vitro (2016) 31:12–22. 10.1016/j.tiv.2015.11.003 26551149

[23] Fromen CA Kelley WJ Fish MB Adili R Nobl J Hoenerhoff MJ Neutrophil–Particle Interactions in Blood Circulation Drive Particle Clearance and Alter Neutrophil Responses in Acute Inflammation. ACS Nano (2017) 11:10797–807. 10.1021/acsnano.7b03190 PMC5709153 29028303

[24] Kelley WJ Onyskiw PJ Fromen CA Eniola-Adefeso O Model Particulate Drug Carriers Modulate Leukocyte Adhesion in Human Blood Flows. ACS Biomater Sci Eng (2019) 5:6530–40. 10.1021/acsbiomaterials.9b01289 PMC9588176 33417805

[25] Macdougall I Strauss W McLaughlin J Li Z Dellana F Hertel J A randomized comparison of ferumoxytol and iron sucrose for treating iron deficiency anemia in patients with CKD. Clin J Am Soc Nephrol (2014) 4:705–12. 10.2215/CJN.05320513 PMC3974353 24458078

[26] Coyne DW Ferumoxytol for treatment of iron deficiency anemia in patients with chronic kidney disease. Expert Opin Pharmacother (2009) 10(15):2563–8. 10.1517/14656560903224998 19708851

[27] Foster GA Gower RM Stanhope KL Havel PJ Simon SI Armstrong EJ On-chip phenotypic analysis of inflammatory monocytes in atherogenesis and myocardial infarction. Proc Natl Acad Sci USA (2013) 110:13944–9. 10.1073/pnas.1300651110 PMC3752270 23918401

[28] Lum AF Green CE Lee GR Staunton DE Simon SI Dynamic regulation of LFA-1 activation and neutrophil arrest on intercellular adhesion molecule 1 (ICAM-1) in shear flow. J Biol Chem (2002) 277:20660–70. 10.1074/jbc.M202223200 11929876

[29] Miralda I Uriarte SM McLeish KR Multiple phenotypic changes define neutrophil priming. Front Cell Infect Microbiol (2017) 7:217. 10.3389/fcimb.2017.00217 28611952 PMC5447094

[30] Rose JJ Foley JF Murphy PM Venkatesan S On the mechanism and significance of ligand-induced internalization of human neutrophil chemokine receptors CXCR1 and CXCR2. J Biol Chem (2004) 279:24372– 24386. 10.1074/jbc.M401364200 15028716

[31] Zhu L He P fMLP-stimulated release of reactive oxygen species from adherent leukocytes increases microvessel permeability. Am J Physiol Heart Circ Physiol (2006) 290:H365–72. 10.1152/ajpheart.00812.2005 16155097

[32] Guichard C Pedruzzi E Dewas C Fay M Pouzet C Bens M Interleukin-8-induced priming of neutrophil oxidative burst requires sequential recruitment of NADPH oxidase components into lipid rafts. J Biol Chem (2005) 280:37021–32. 10.1074/jbc.M506594200 16115878

[33] Schaff UY Xing MM Lin KK Pan N Jeon NL Simon SI Vascular mimetics based on microfluidics for imaging the leukocyte–endothelial inflammatory response. Lab Chip (2007) 7:448–56. 10.1039/b617915k 17389960

[34] Morikis VA Chase S Wun T Chaikof EL Magnani JL Simon SI; Selectin catch-bonds mechanotransduce integrin activation and neutrophil arrest on inflamed endothelium under shear flow. Blood (2017) 130:2101–10. 10.1182/blood-2017-05-783027 PMC5680610 28811304

[35] Schaff UY Yamayoshi I Tse T Griffin D Kibathi L Simon SI Calcium flux in neutrophils synchronizes beta2 integrin adhesive and signaling events that guide inflammatory recruitment. Ann BioMed Eng (2008) 36:632–46. 10.1007/s10439-008-9453-8 PMC2668576 18278555

[36] Immler R Simon SI Sperandio M Calcium signalling and related ion channels in neutrophil recruitment and function. Eur J Clin Invest (2018) 48(Suppl. 2):e12964. 10.1111/eci.12964 29873837 PMC6221920

[37] Anderson R Visser SS Ramafi G Theron AJ Accelerated resequestration of cytosolic calcium and suppression of the pro-inflammatory activities of human neutrophils by CGS 21680 in vitro. Br J Pharmacol (2000) 130:717–24. 10.1038/sj.bjp.0703344 PMC1572119 10864876

[38] Morikis VA Simon SI Neutrophil mechanosignaling promotes integrin engagement with endothelial cells and motility within inflamed vessels. Front Immunol (2018) 9:2774. 10.3389/fimmu.2018.02774 30546362 PMC6279920

[39] Simon SI Hu Y Vestweber D Smith CW Neutrophil tethering on E-selectin activates beta 2 integrin binding to ICAM-1 through a mitogen-activated protein kinase signal transduction pathway. J Immunol (2000) 164:4348–58. 10.4049/jimmunol.164.8.4348 10754335

[40] Bilyy R Unterweger H Weigel B Dumych T Paryzhak S Vovk V Inert Coats of Magnetic Nanoparticles Prevent Formation of Occlusive Intravascular Co-aggregates With Neutrophil Extracellular Traps. Front Immunol (2018) 9:2266. 10.3389/fimmu.2018.02266 30333831 PMC6176021

[41] Cappenberg A Margraf A Thomas K Bardel B McCreedy DA Van Marck V L-selectin shedding affects bacterial clearance in the lung: a new regulatory pathway for integrin outside-in signaling. Blood (2019) 134:1445–57. 10.1182/blood.2019000685 PMC6839956 31366620

[42] Mueller H Stadtmann A Van Aken H Hirsch E Wang D Ley K Tyrosine kinase Btk regulates E-selectin-mediated integrin activation and neutrophil recruitment by controlling phospholipase C (PLC) gamma2 and PI3Kgamma pathways. Blood (2010) 115:3118–27. 10.1182/blood-2009-11-254185 PMC2858472 20167705

