# Supplementary figures and images for: Neutrophil Inflammatory Response Is Downregulated by Uptake of Superparamagnetic Iron Oxide Nanoparticle Therapeutics
Source: Front Immunol. 2020 Dec 9;11:571489. doi: 10.3389/fimmu.2020.571489 (PMC7757401; doi:10.3389/fimmu.2020.571489)

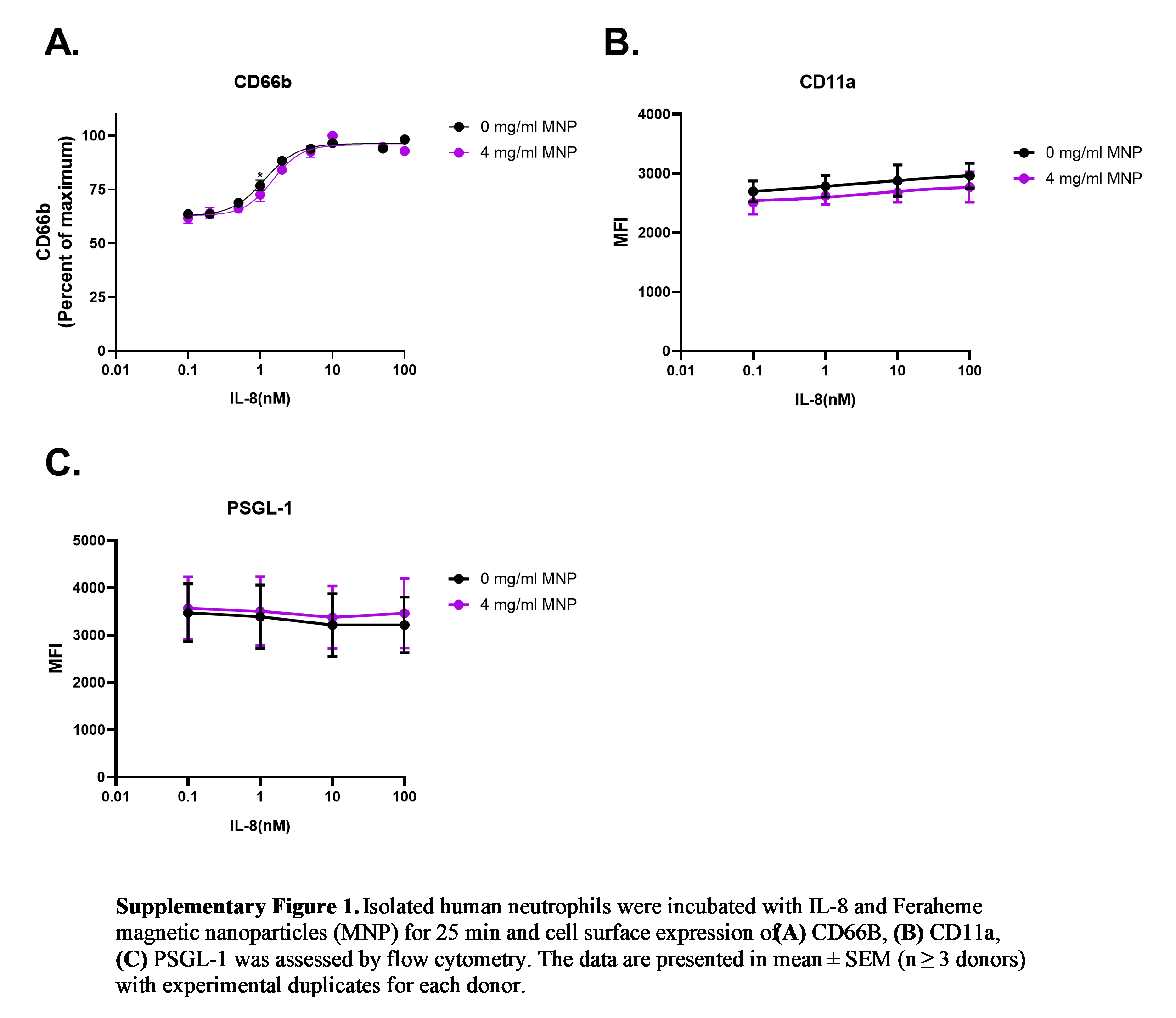

Supplement: Supplementary file 1 [file Image_1.tif]
